# TRPC5 is essential in endothelium-dependent contraction of aorta from diet-induced obese mice

**DOI:** 10.1016/j.fmre.2022.01.017

**Published:** 2022-01-31

**Authors:** Yifei Zhu, Sheng Wang, Yuan Chu, Ka Zhang, Xin Wen, Lei Feng, Fan Yu, Xin Ma

**Affiliations:** Wuxi School of Medicine, Jiangnan University, Wuxi 214000, China

**Keywords:** Transient receptor potential channel canonical family member 5 (TRPC5), Endothelium-dependent contraction, Vascular function, Diet-induced obesity

## Abstract

The role of the Ca^2+^-permeable ion channel TRPC5 in regulating vasocontraction in obesity is poorly understood. Here, we investigated whether TRPC5 contributes to vascular dysfunction in obesity by promoting endothelium-dependent contraction *via* activation of cytosolic phospholipase A2 (cPLA_2_) in the aortic endothelial cells of obese mice. Acetylcholine-induced endothelium-dependent relaxation and contraction in the aorta were measured using wire myography. PLA_2_ activity was measured by the fluorogenic PLA_2_ substrate Bis-BODIPY™ FL C_11_-PC. The intracellular Ca^2+^ level in response to acetylcholine was measured by Fluo-4 fluorescence. Endothelium-derived contracting factors were assessed by enzyme immunoassay. Diet-induced obesity (DIO) attenuated endothelium-dependent vasodilation, enhanced endothelium-dependent contraction (EDC), and increased the expression of TRPC5 in the mouse aorta. Activation of TRPC5 promoted EDC in the wild-type mouse aorta, whereas pharmacological inhibition and genetic knockout of TRPC5 decreased EDC in the DIO mouse aorta. Moreover, cPLA_2_ phosphorylation and activity were higher in aortic endothelial cells from DIO mice, and this was attenuated by inhibition and knockout of TRPC5. Cyclooxygenase 2 (COX-2) expression was increased in DIO mouse endothelium and was decreased by a TRPC5 inhibitor and knockout of TRPC5. Release of prostaglandins F_2α_ (PGF_2α_) and E_2_ (PGE_2_) was involved in TRPC5-regulated EDC in DIO mice. This study demonstrated that TRPC5 contributes to endothelial and vascular dysfunction and is involved in EDC through activation of cPLA_2_ and enhanced COX-2-PGF_2α_/PGE_2_ levels in DIO mice.

## ☆

**Figure:**
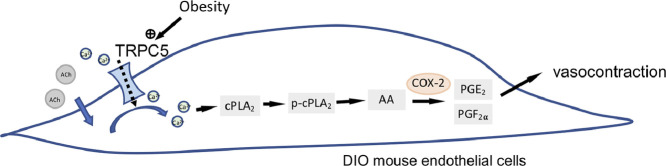

## 1 Introduction

The mechanisms by which high-fat diet-induced obesity (DIO) increases the risk of cardiovascular disease are not completely understood. However, it is known that DIO is related to endothelial dysfunction and impaired endothelium-dependent vascular tone [1] which lead to the development of atherosclerosis [2]. Identifying the molecular mechanisms of obesity-induced endothelial dysfunction will help to establish new strategies for therapy.

Endothelium regulates vascular tone by keeping the balance between beneficial endothelium-derived relaxing factors (EDRFs) and harmful endothelium-derived contracting factors (EDCFs) in response to substances in the blood and the shear stress of blood flow [3]. Endothelium-dependent contraction (EDC) induced by EDCFs is usually augmented under disease conditions such as hypertension and hyperlipidemia [4]. EDC can be induced by acetylcholine (ACh) in arteries pre-treated with a nitric oxide synthase inhibitor due to extracellular Ca^2+^ influx *via* nonselective cation channels [5]. The underlying mechanisms of intracellular Ca^2+^ ([Ca^2+^]_i_) signal regulation associated with ACh need further study. EDCFs such as vasoconstrictor prostanoids cause EDC associated with an elevated endothelial [Ca^2+^]_i_ level [6]. The [Ca^2+^]_i_ rise triggers cytosolic phospholipase A2 (cPLA_2_), which turns arachidonic acid into prostanoids *via* cyclooxygenase [6]. A recent study has shown the involvement of the transient receptor potential vanilloid 4 channel in EDCFs release in the hypertensive mouse aorta [3].

Transient receptor potential channel canonical family member 5 (TRPC5) is a Ca^2+^-permeable channel expressed in the cardiovascular system that plays a key role in pressure sensing by aortic baroreceptors [7]. A previous study has shown that TRPC5 is able to regulate angiogenesis under hypoxia-ischemia [8]. Recent studies have indicated its involvement in endothelial cell senescence and carotid artery contraction under physiological conditions [9,10]. However, it is not clear whether TRPC5 is involved in regulating EDC in the aorta of obese mice, and the underlying mechanisms remain elusive.

In the present study, we hypothesized that TRPC5 contributes to vascular dysfunction in obesity by promoting EDC. We demonstrated that TRPC5 enhances EDC in the DIO mouse aorta through the activation of cytosolic cPLA_2_ in endothelial cells.

## 2 Material and methods/experiment

### 2.1 Animals

TRPC5-knockout (TRPC5^−/−^) and wild-type (WT) mice on the C57BL/6J background were a kind gift from Professor David E. Clapham (Harvard Medical School, USA) and maintained in Jiangnan University. All mice were kept at 22, 23 °C with a 12 h light/dark cycle. At the age of 6–8 weeks, TRPC5^−/−^ and WT mice were fed standard chow (normal-fat diet, NFD) or a high-fat diet (HFD, 45% kCal from fat; TP23200, Trophic Animal Feed High-tech Co., Ltd., Nantong, China) for >3 months to develop DIO. Metabolic parameters, including body weight, fat pad mass, and blood glucose, as well as the lipid profile, were confirmed in HFD and NFD mice (Table S1). Age- and sex-matched TRPC5^−/−^ and WT littermates were selected by an established polymerase chain reaction genotyping method (Fig. S1) for this study. CO_2_ inhalation was used for euthanasia. All animal experiments were performed in accordance with the guidelines issued by the National Institutes of Health, USA, and approved by the Animal Experiments Ethics Committee of Jiangnan University (approval number: JN. 20201115t0121231[298]).

### 2.2 Primary cells

Primary mouse aortic endothelial cells (MAoECs) from NFD and HFD mice were cultured in EC medium (ScienCell, Carlsbad, CA, USA). Confluent cells after <3 passages were used. To obtain the cells, mice were euthanized with CO_2_, and the aorta was acutely dissected, placed in sterile PBS, and digested in 0.02% collagenase type 1A for 25 min at 37 °C. The digested tissue was centrifuged at 1200 g for 5 min and the pelleted cells were re-suspended and cultured at 37 °C with 5% CO_2_. Non-adherent cells were removed 2 h later.

### 2.3 Wire myography

The aorta was removed to cold Krebs solution, divided into 2-mm segments, and mounted in a myograph (model 610M, Danish Myo Technology, Aarhus, Denmark). The chamber contained 95% O_2_ and 5% CO_2_ and was maintained at 37 °C. After equilibration at 0 mN for 30 min, a ring was stretched to an optimal baseline force of 3 mN for 30 min. KCl (60 mmol/L) was applied to confirm the contractility of the artery. For EDR measurement, aortic rings were pre-contracted with 1 μmol/L phenylephrine (phe) for ∼5 min before exposure to progressively increasing concentrations of ACh. In EDC experiments, the segments were pre-incubated with L-NAME (100 μmol/L) for 30 min before application of ACh. Aortas were pre-treated with AM237, clemizole (Aladdin), methylarachidonyl fluorophosphonate (MAFP, Cayman), NS-398 (Sigma), or VAS-2870 (Sigma) as necessary. AM237 efficiency was confirmed by patch clamp (Fig. S2) as previously described [11]. In some experiments, the endothelium was mechanically denuded by rubbing the lumen gently with a steel wire. Successful removal was confirmed by the lack of a relaxant response to bradykinin (100 nmol/L).

### 2.4 Western blot

Aortic endothelial cells were lysed in lysis buffer (P0013C, Beyotime) on ice with protease and phosphatase inhibitors (Beyotime). The lysates were collected by centrifugation. The BCA assay was used for protein quantification. Proteins were electrophoresed on 10% SDS-polyacrylamide gels and then transferred to polyvinylidene fluoride membranes (Millipore Corp., Bedford, MA, USA). Skim milk powder (5%) was used for blocking. The membranes were incubated overnight at 4 °C with the primary antibodies anti-TRPC5 (1:200, Proteintech), anti-COX-1 (1:200, Abcam), anti-COX-2 (1:2000, Abcam), anti-cPLA_2_ (1:200, Santa Cruz), anti-p-cPLA_2_ (1:1000, Signalway Antibody), and anti-GAPDH (1:1000, Santa Cruz) followed by horseradish peroxidase-conjugated secondary antibody (mouse, 1:10,000; rabbit, 1:5000, Beyotime) at room temperature for 2 h. ImageJ was used for band intensity analysis. The specificity of TRPC5 antibody was confirmed (Fig. S3).

### 2.5 Immunofluorescence

Frozen sections of the mouse aorta were fixed in 4% paraformaldehyde (Sigma-Aldrich), washed with PBS, blocked with 5% BSA, and incubated with anti-CD31 (1:200, Abcam) and anti-TRPC5 (1:200, Proteintech) antibodies overnight at 4 °C followed by secondary antibodies (AlexaFluor 546 anti-rabbit and AlexaFluor 647 anti-mouse, 1:200) at room temperature for 2 h. Nuclei were stained with DAPI. Images were captured on a Zeiss LSM 880 confocal microscope.

### 2.6 PLA_2_ activity

A fluorogenic PLA_2_ substrate (Bis-BODIPY™ FL C_11_-PC) (Thermo Fisher Scientific) was used for PLA_2_ activity measurement in the *en-face* aorta. Aortas were incubated with Bis-BODIPY™ FL C_11_-PC in normal physiological saline containing (in mmol/L): 140 NaCl, 5 KCl, 1 CaCl_2_, 1 MgCl_2_, 10 glucose, 5 HEPES, and 2% BSA (pH 7.4) for 20 min at 37 °C in the dark. An Andor confocal microscope (Dragonfly 200) was used to capture images with 480 nm excitation and 520 nm emission. The fluorescence intensity was analyzed by ImageJ. Aortas were pretreated with activator or inhibitor for 30 min if necessary, and then stimulated with ACh for 10 min.

### 2.7 Ca^2+^ measurement

Ca^2+^ imaging was performed in *en-face*, fluo-4-loaded (1:1000, Thermo) aortas in normal physiological saline containing (in mmol/L) 140 NaCl, 5 KCl, 1 CaCl_2_, 1 MgCl_2_, 10 glucose, 5 HEPES, and 2% BSA (pH 7.4) for 15 min at 37 °C in the dark. Live-cell images of the [Ca^2+^]_i_ were acquired and analyzed using the Andor confocal microscope (Dragonfly 200). [Ca^2+^]_i_ was quantified as the ratio of real-time fluorescence (F1) to the intensity immediately after application of ACh (10 μmol/L). We evaluated 5–15 randomly-selected cells in each replication.

### 2.8 Enzyme immunoassay (EIA)

Aortic rings were isolated from NFD and HFD mice and incubated with AM237, clemizole, MAFP, NS-398, or VAS-2870 as necessary. ACh (10 μmol/L for 5–10 min) was used to stimulate the production of contraction factors. Then, the tissue was removed and the solution was centrifuged at 5000 rpm for 5 min at 4 °C. The solutions were frozen and stored at –80 °C until EIA assay. PGF_2α_, 8-isoprostanes, PGE_2_, PGI_2_, and PGD_2_ were measured using EIA kits (Meimian, China) according to the manufacturer's instructions.

### 2.9 Statistics

Data are represented as the mean ± standard error of the mean (SEM). Statistics were analyzed using GraphPad Prism 6.0 software. Concentration-response curves were constructed by two-way analysis of variance with repeated measures followed by the Bonferroni test. Comparisons between two groups were analyzed by Student's unpaired two-tailed *t*-test or the Mann-Wallis test. Differences among three or more groups were measured by one-way analysis of variance followed by Dunnett's or Tukey's multiple comparison test or Kruskal-Wallis and Dunn's *post hoc* non-parametric test. *P*-values <0.05 were considered to be significantly different.

## 3 Results

### 3.1 Role of diet-induced obesity in ACh-induced relaxation and contraction of mouse aorta

**Fig. 1:**
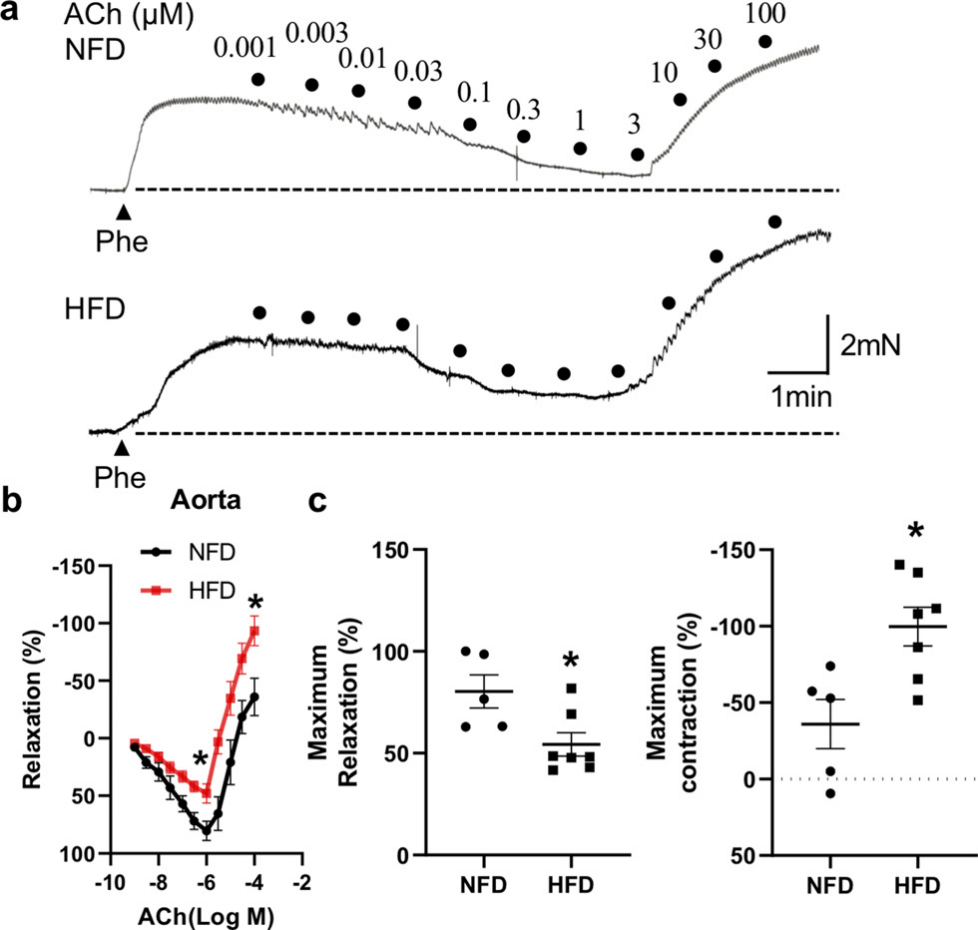
**Acetylcholine (ACh)-induced relaxation followed by contraction in the normal-fat diet (NFD) control and high-fat diet (HFD)-induced obese mouse aorta.** (a–c) Original recordings (a) and data summary (b and c) of ACh-induced concentration-dependent vasodilation and contraction in phenylephrine (Phe)-preconstricted NFD (*n* = 5) and HFD (*n* = 7) mouse aortas. Mean ± SEM; *P < 0.05 *vs* NFD, b, two-way ANOVA followed by Bonferroni test; c, Student's unpaired two-tailed *t* test.

A wire myograph was used to measure vascular tone in the mouse aorta. In the phe-precontracted aorta, ACh induced a concentration-dependent relaxation in the range of 0.001–0.3 μmol/L in both NFD control and DIO mice in the absence of L-NAME. Further increases of ACh concentration from 1 to 100 μmol/L led to contraction in the control and DIO mouse aorta (Fig. 1a). The magnitude of ACh-induced relaxation was significantly lower and ACh-induced contraction was stronger in the DIO mouse aorta than in the NFD control (Fig. 1a–c). These results suggested that DIO is associated with vascular dysfunction.

### 3.2 Activation of TRPC5 enhances endothelium-dependent vasoconstriction in mouse aorta

**Fig. 2:**
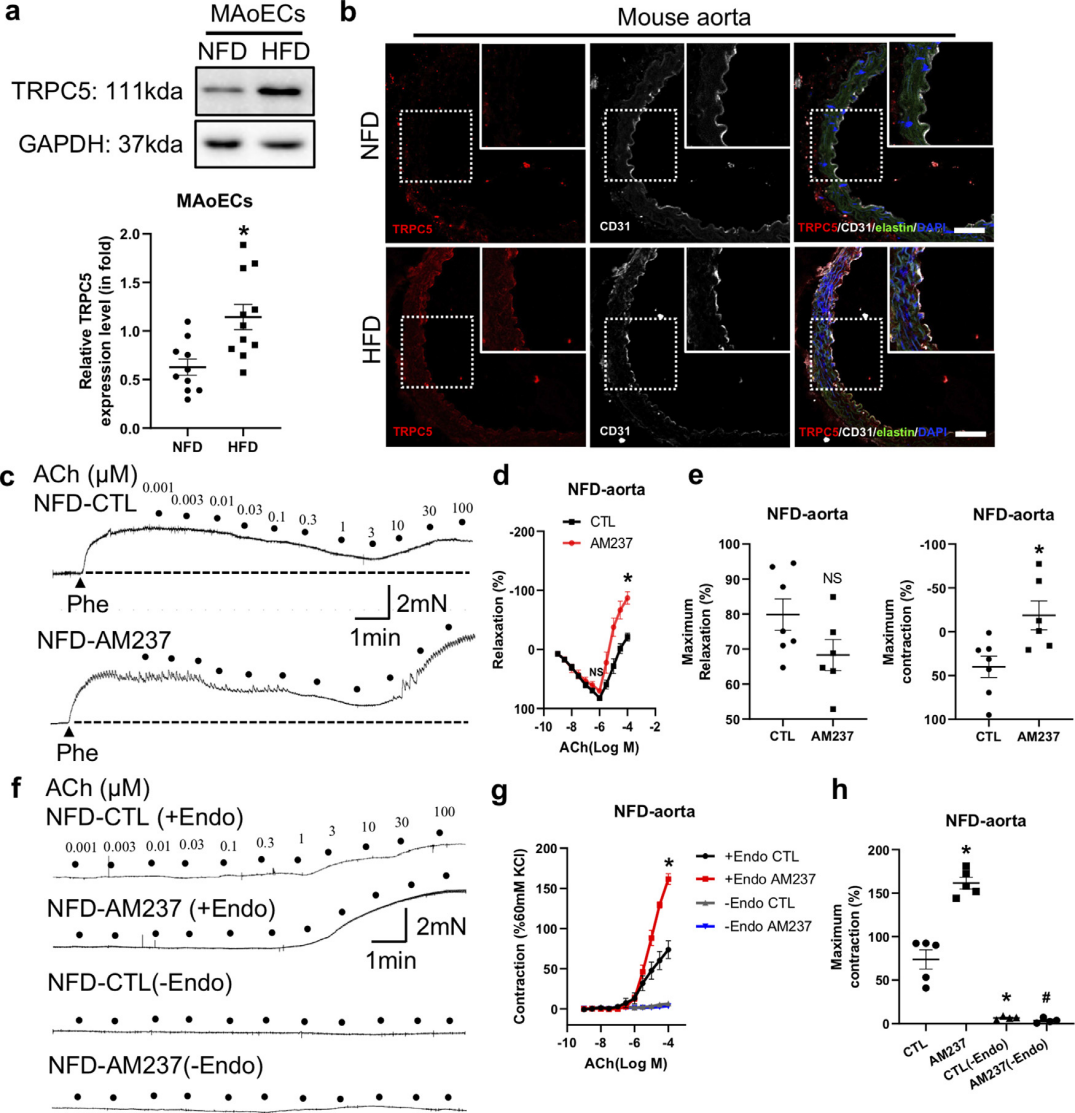
**Effect of the TRPC5 activator AM237 on acetylcholine (ACh)-induced relaxation and contraction in the normal-fat diet (NFD) mouse aorta.** (a) TRPC5 protein expression in mouse aortic endothelial cells (MAoECs) from NFD (*n* = 10) and high-fat diet (HFD)-induced obese (*n* = 11) mice. (b) Co-immunostaining of TRPC5 and CD31 in mouse aortic rings (scale bar, 50 μm). (c–e) Representative traces (c) and summary data (d, e) of phenylephrine (Phe)-precontracted ACh-induced relaxation followed by contraction at high concentrations in NFD (*n* = 7) and AM237 (100 nmol/L)-pretreated NFD (*n* = 6) aortas. (f–h) Original recordings (f) and data summary (g, h) showing the effect of AM237 (100 nmol/L) on ACh-induced contraction in the NFD aorta with or without endothelium (+endo CTL, *n* = 5; +endo AM237, *n* = 5; –endo CTL, *n* = 4; –endo AM237, *n* = 4). Mean ± SEM; A, *P < 0.05 *vs* NFD, Student's unpaired two-tailed *t* test; d, *P < 0.05, NS, no significant difference *vs* NFD, two-way ANOVA followed by Bonferroni test; e, *P < 0.05, NS, no significant difference *vs* CTL, Student's unpaired two-tailed *t* test; g, *P < 0.05 *vs* +endo CTL, two-way ANOVA followed by Bonferroni test; h, *P < 0.05 *vs* CTL, ^#^*P* < 0.05 *vs* AM237, one-way ANOVA followed by Turkey's multiple comparisons test.

Western blot analysis and immunostaining were used to investigate the expression of TRPC5 in endothelial cells from the NFD control and DIO mouse aorta. As expected, TRPC5 was expressed in these cells. Moreover, the expression level of TRPC5 was much higher in DIO endothelial cells (Fig. 2a and b). To investigate whether TRPC5 is involved in the decreased vasodilation and enhanced vasocontraction, we assessed the response of vascular tone to AM237, a potent and selective TRPC5 activator [11], by measuring ACh-induced concentration-dependent vasodilation and contraction in the NFD control mouse aorta preconstricted with phe. The results showed that ACh-induced relaxation from 0.001 to 0.3 μmol/L did not change in aortas treated with 100 nmol/L AM237. However, further increases in ACh concentration induced contraction that was significantly enhanced in AM237-treated NFD control aortas (Fig. 2c–e). We then investigated the effect of AM237 on ACh-induced vasoconstriction in the presence of L-NAME (100 μmol/L) without phe pretreatment. In this case, the magnitude of ACh-induced contraction was significantly higher in the AM237-treated intact aorta, while removal of the endothelium inhibited the ACh-induced contraction with or without AM237 (Fig. 2f–h). These results showed that TRPC5 activation increases ACh-induced EDC in the mouse aorta.

### 3.3 TRPC5 regulates endothelium-dependent contraction in aorta from obese mice

**Fig. 3:**
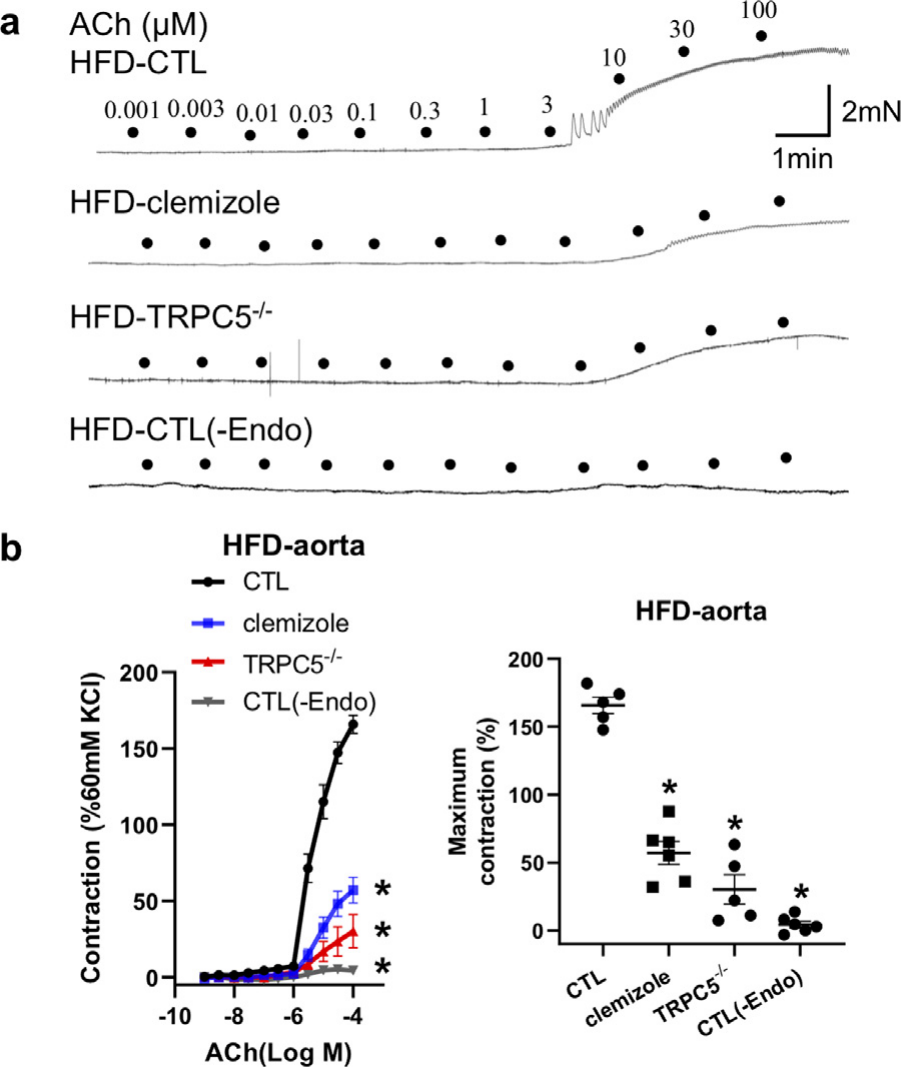
**Effect of TRPC5 inhibition on acetylcholine (ACh)-induced vasoconstriction in the high-fat diet (HFD)-induced obese mouse aorta.** (a, b) Representative traces (a) and data summary (b) showing ACh-induced contraction is attenuated by the TRPC5 inhibitor clemizole (20 μmol/L), knockout of TRPC5, and the removal of endothelium (CTL, *n* = 5; clemizole, *n* = 6; TRPC5^−/−^, *n* = 5; CTL(–Endo), *n* = 6; mean ± SEM; b, left, *P < 0.05 *vs* CTL, two-way ANOVA followed by Bonferroni test; right, *P < 0.05 *vs* CTL, one-way ANOVA followed by Dunnett's multiple comparisons test).

We used TRPC5^−/−^ mice and clemizole, a TRPC5 inhibitor, to study the role of TRPC5 in DIO mouse EDC. Wire myography was used to measure vascular tone. We found that ACh-induced contraction in the presence of L-NAME was attenuated by the inhibitor and by knockout of TRPC5 (Fig. 3a and b). As expected, removal of endothelium abolished the ACh-elicited contraction. These results demonstrated a vital role of TRPC5 in EDC of the obese mouse aorta.

### 3.4 Role of cPLA_2_ activation in TRPC5-regulated contraction of DIO mouse aorta

**Fig. 4:**
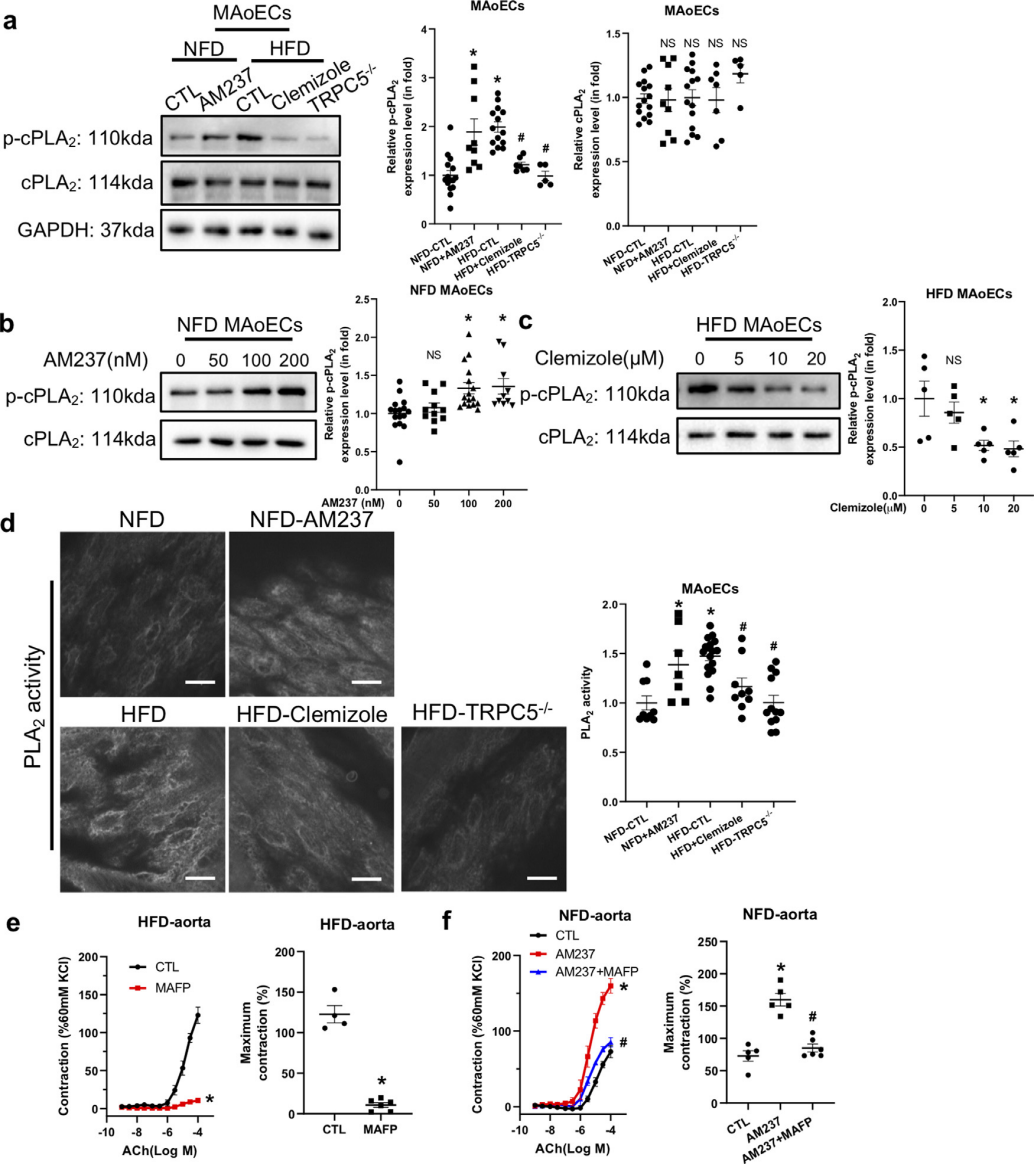
**TRPC5 regulates contractions*****via*****cytosolic phospholipase A**_**2**_**(cPLA**_**2**_**) in the high-fat diet (HFD)-induced obese mouse aorta.** (a) Western blots and analysis of cPLA_2_ and phosphorylated cPLA_2_ (p-cPLA_2_) expression in normal-fat diet (NFD, *n* = 15), AM237 (100 nmol/L)-treated NFD (*n* = 9), HFD-induced obese (*n* = 14), clemizole (20 μmol/L)-treated HFD (*n* = 7), and TRPC5^−/−^-HFD (*n* = 5) mouse aortic endothelial cells (MAoECs). (b) Dose-dependent effect of AM237 on p-cPLA_2_ levels in NFD MAoECs. AM237 (nmol/L), 0, *n* = 16; 50, *n* = 11; 100, *n* = 16; 200, *n* = 10. (c) Dose-dependent effect of clemizole on p-cPLA_2_ levels in HFD MAoECs (*n* = 5). (d) Representative fluorescence images of the Bis-BODIPY™ FL C_11_-PC stained *en-face* aorta and analysis of PLA_2_ activity in endothelial cells of the NFD (*n* = 9), AM237 (100 nmol/L)-pretreated NFD (*n* = 7), HFD (*n* = 17), clemizole (20 μmol/L)-treated HFD (*n* = 9), and TRPC5^−/−^ HFD (*n* = 12) mouse aorta (scale bars, 10 μm). (e) Acetylcholine (ACh)-induced contraction in HFD (*n* = 4) and MAFP (10 μmol/L)-treated HFD (*n* = 6) mouse aortic rings. (f) ACh-induced contraction in the NFD (*n* = 5), AM237 (100 nmol/L)-pretreated NFD (*n* = 5), and AM237 (100 nmol/L) +MAFP (30 μmol/L)-pretreated NFD (*n* = 6) mouse aorta. Mean ± SEM; a, *P < 0.05 vs NFD, ^#^*P* < 0.05 *vs* HFD, NS, no significant difference, Kruskal-Wallis and Dunn's *post hoc* non-parametric test (p-cPLA_2_) and one-way ANOVA followed by Turkey's multiple comparisons test (cPLA_2_); b, *P < 0.05, NS, no significant difference *vs* no AM237, one-way ANOVA followed by Dunnett's multiple comparisons test; c, *P < 0.05, NS, no significant difference *vs* no clemizole, one-way ANOVA followed by Dunnett's multiple comparisons test; d, *P < 0.05 *vs* NFD, ^#^*P* < 0.05 vs HFD, one-way ANOVA followed by Turkey's multiple comparisons test; e, *P < 0.05 *vs* CTL, two-way ANOVA followed by Bonferroni test (left) and Student's unpaired two-tailed *t* test (right); f, *P < 0.05 *vs* CTL, ^#^*P* < 0.05 vs AM237, two-way ANOVA followed by Bonferroni test (left) and one-way ANOVA followed by Turkey's multiple comparisons test (right).

To investigate the mechanism underlying TRPC5-regulated vasocontraction, we studied cytosolic phospholipase A2 (cPLA_2_), a Ca^2+^-sensitive vasocontractile agonist [3]. Western blot analysis and enzyme immunoassays showed that, compared to the NFD control, cPLA_2_ phosphorylation and PLA_2_ activity was increased in DIO MAoECs while the cPLA_2_ level did not change (Fig. 4a and d). Importantly, AM237 treatment significantly increased cPLA_2_ phosphorylation in a dose-dependent manner and enhanced PLA_2_ activity but did not alter the expression of cPLA_2_ in NFD control MAoECs (Fig. 4a, b and d). As expected, both pharmacological inhibition of TRPC5 by clemizole and knockout of TRPC5 significantly attenuated the cPLA_2_ phosphorylation and PLA_2_ activity in DIO MAoECs (Fig. 4a and d). We also found that clemizole decreased cPLA_2_ phosphorylation in a concentration-dependent manner (Fig. 4c). MAFP (10 μmol/L), an inhibitor of cPLA_2_, significantly attenuated the ACh-induced vasocontraction in both the intact DIO and AM237-treated NFD control mouse aorta (Fig. 4e and f).

**Fig. 5:**
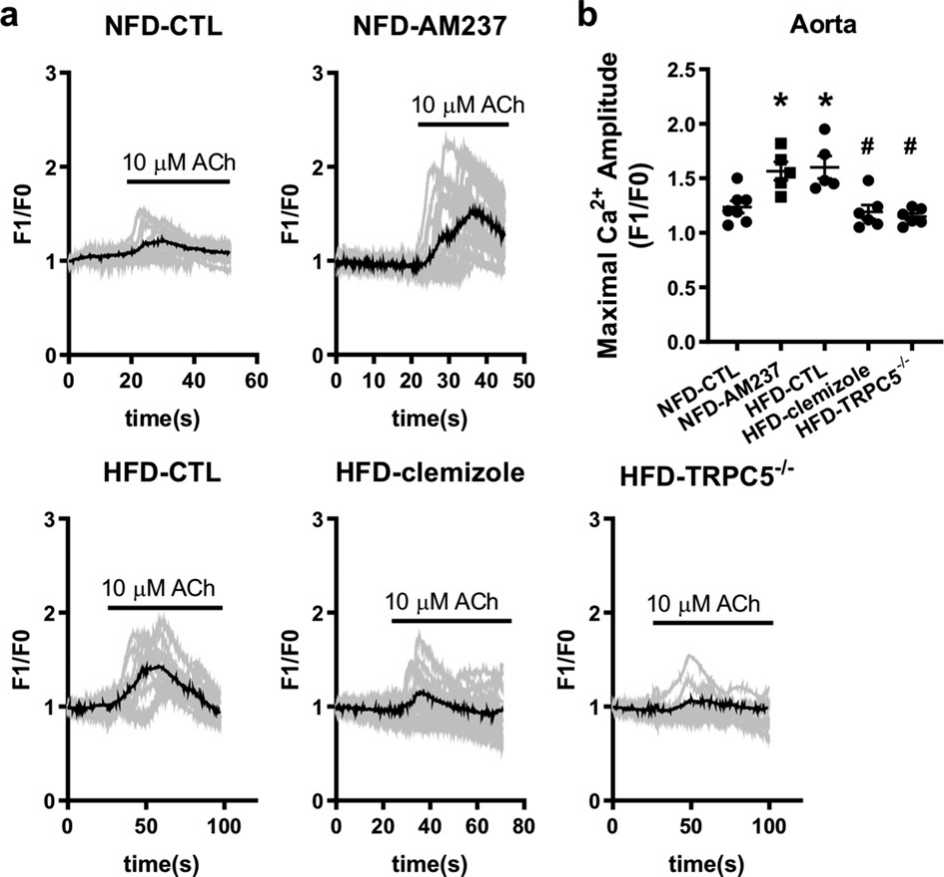
**TRPC5 contributes to acetylcholine (ACh)-induced Ca**^**2+**^**entry into endothelial cells of high-fat diet (HFD)-induced obese mouse aortas.** (a, b) Representative traces (a) and data summary (b) showing an ACh (10 μmol/L)-induced [Ca^2+^]_i_ rise in aortic endothelial cells from normal-fat diet (NFD) and HFD mice (NFD, *n* = 7; NFD-AM237 (100 nmol/L), *n* = 5; HFD, *n* = 5; HFD-clemizole (20 μmol/L), *n* = 6; HFD-TRPC5^−/−^, *n* = 6; mean ± SEM; *P < 0.05 *vs* NFD, ^#^*P* < 0.05 *vs* HFD, one-way ANOVA followed by Turkey's multiple comparisons test).

Notably, the Ca^2+^ influx induced by 10 μmol/L ACh was much higher in DIO MAoECs than in the NFD control (Fig. 5a and b). Moreover, AM237 treatment enhanced the ACh-induced Ca^2+^ entry in NFD control MAoECs (Fig. 5a and b). As expected, the [Ca^2+^]_i_ was markedly reduced in clemizole-treated DIO and TRPC5^−/−^ DIO MAoECs (Fig. 5a and b).

Together, these results demonstrated that TRPC5-regulated vascular contraction is associated with an intracellular Ca^2+^ rise and cPLA_2_ activation.

### 3.5 Role of COX-2 in TRPC5-regulated vasoconstriction in the DIO mouse

**Fig. 6:**
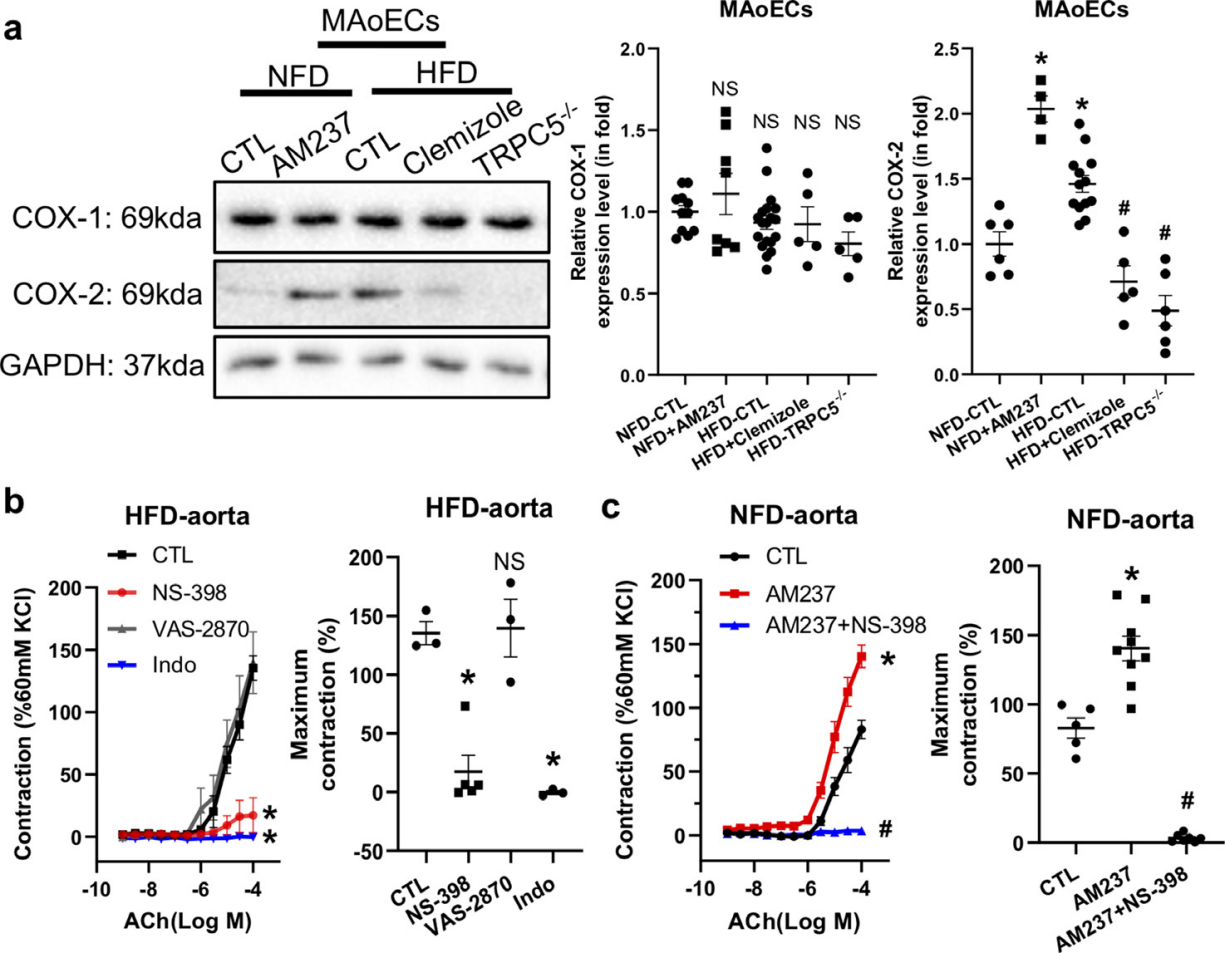
**Role of COX-2 in TRPC5-regulated vasoconstriction in the mouse aorta.** (a) Western blots and analysis of COX-1 and COX-2 expression in normal-fat diet (NFD, *n* = 5), AM237 (100 nmol/L) pre-treated NFD (*n* = 5), high-fat diet (HFD, *n* = 5), clemizole (20 μmol/L) pre-treated HFD (*n* = 5), and TRPC5^−/−^ HFD (*n* = 5) mouse aortic endothelial cells (MAoECs). (b) Effect of the COX inhibitors NS-398 (3 μmol/L) (*n* = 5), VAS-2870 (30 μmol/L) (*n* = 3), and indomethacin (indo, 1 μmol/L, *n* = 3) on acetylcholine (ACh)-induced contraction in the HFD mouse aorta (CTL, *n* = 3). (c) Effect of the COX-2 inhibitor NS-398 (3 μmol/L) on ACh-induced contraction in the AM237 (100 nmol/L)-pretreated NFD mouse aorta (CTL, *n* = 5; AM237, *n* = 7; AM237+NS-398, *n* = 7). Mean ± SEM; a, *P < 0.05 *vs* NFD, ^#^*P* < 0.05 *vs* HFD, NS, no significant difference, Kruskal-Wallis and Dunn's *post hoc* non-parametric test (COX-1) and one-way ANOVA followed by Turkey's multiple comparisons test (COX-2); b, *P < 0.05, NS, no significant difference *vs* CTL, two-way ANOVA followed by Bonferroni test (left) and one-way ANOVA followed by Dunnett's multiple comparisons test (right); c, *P < 0.05 *vs* CTL, ^#^*P* < 0.05 *vs* AM237, two-way ANOVA followed by Bonferroni test (left) and one-way ANOVA followed by Turkey's multiple comparisons test (right) .

We then investigated the involvement of COX in the TRPC5-cPLA_2_-regulated vasocontraction in obesity (Fig. 6). Immunoblot data showed that COX-2 expression was much higher in DIO MAoECs than in the NFD control while there was no difference of COX-1 expression between the two groups (Fig. 6a). Moreover, activation of TRPC5 by AM237 (100 nmol/L) markedly increased the COX-2 level in NFD control MAoECs (Fig. 6a). As expected, the TRPC5 inhibitor clemizole (20 μmol/L) and knockout of TRPC5 attenuated COX-2 expression in DIO MAoECs (Fig. 6a). In contrast, the COX-1 level was similar in these groups (Fig. 6a). To identify the specific COX isoform involved in TRPC5-regulated vascular contraction in obesity, we exposed aortas to the nonselective COX inhibitor indomethacin (1 μmol/L), the COX-1 selective inhibitor VAS-2870 (30 μmol/L), and the COX-2 selective inhibitor NS-398 (3 μmol/L), before ACh stimulation. We found significant blockade of ACh-induced contraction in the indomethacin- and NS-398 treated DIO mouse aorta, while the COX-1 inhibitor VAS-2870 did not affect ACh-induced contraction (Fig. 6b). Similarly, indomethacin and NS-398 also inhibited the AM237 (100 nmol/L)-elevated contractile response to ACh (3–100 μmol/L) in the NFD control mouse aorta (Fig. 6c).

### 3.6 PGF_2α_ and PGE_2_ as endothelium-derived contracting factors in DIO mouse

**Fig. 7:**
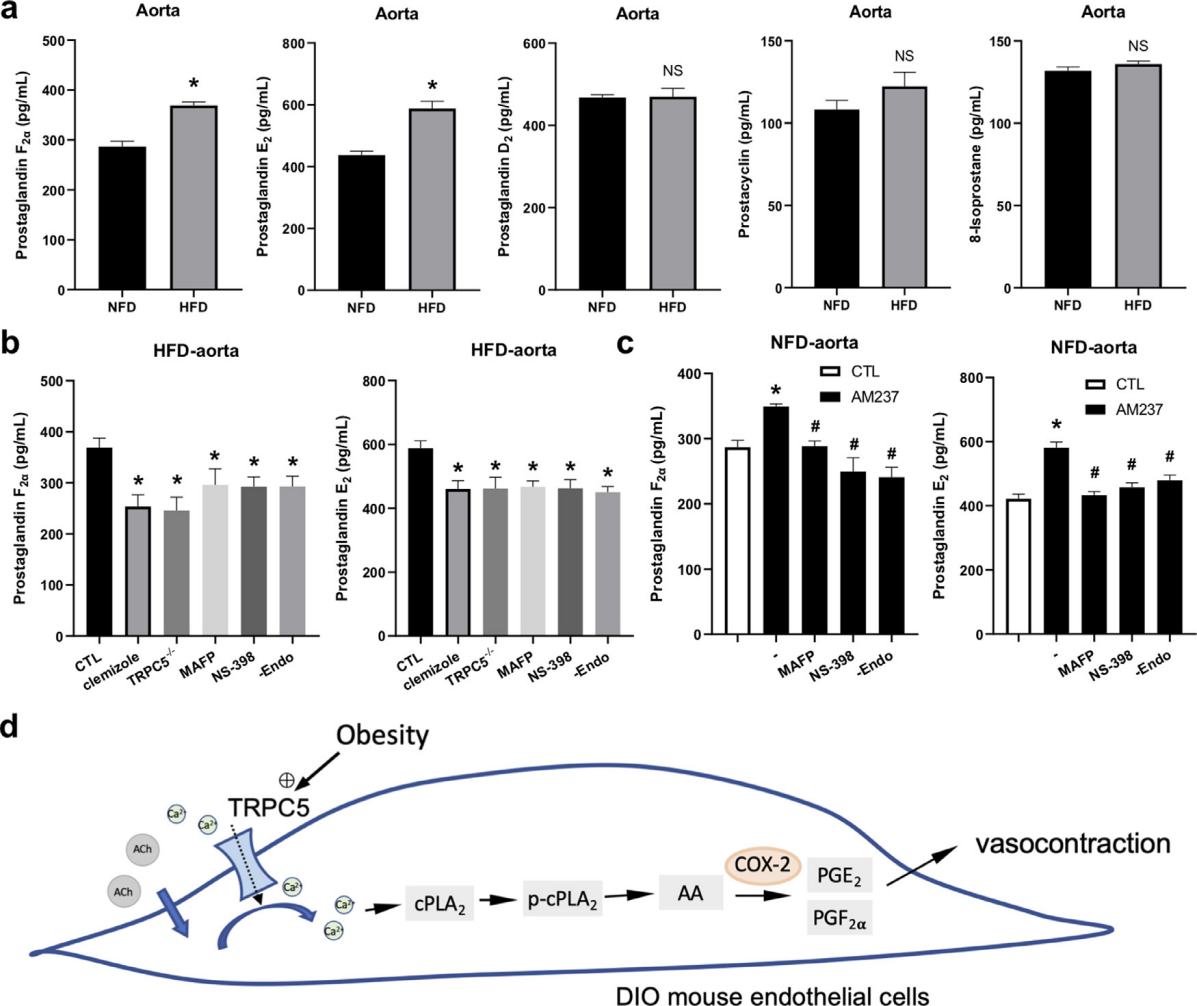
**PGF**_**2α**_**and PGE**_**2**_**are involved in TRPC5-related contraction in the high-fat diet (HFD)-induced obese mouse aorta.** (a) Enzyme immunoassay (EIA) showing the PGF_2α_ (*n* = 7), PGE_2_ (*n* = 7), PGD_2_ (*n* = 5), PGI_2_ (*n* = 5), and 8-isoprostanes (*n* = 6) levels in normal-fat diet (NFD) and HFD mouse aortic rings after exposure to acetylcholine (ACh, 10 μmol/L). (b) Effect of clemizole (20 μmol/L), MAFP (10 μmol/L), NS-398 (3 μmol/L), –endo, and knockout of TRPC5^−/−^ on ACh-induced PGF_2α_ and PGE_2_ release in the ACh-stimulated HFD mouse aorta (PGF_2α_, CTL, *n* = 7; clemizole, *n* = 5; TRPC5^−/−^, *n* = 5; MAFP, *n* = 6; NS-398, *n* = 6; –endo, *n* = 6; PGE_2_, CTL, *n* = 7; clemizole, *n* = 5; TRPC5^−/−^, *n* = 5; MAFP, *n* = 5; NS-398, *n* = 5; –endo, *n* = 5; (c) Effects of AM237 (100 nmol/L), MAFP (10 μmol/L), and NS-398 (3 μmol/L) treatment and the removal of endothelium (–Endo) on EIA for PGF_2α_ and PGE_2_ in the NFD mouse aorta stimulated with ACh (10 μmol/L) (PGF_2α_, CTL, *n* = 7; AM237, *n* = 4; MAFP, *n* = 6; NS-398, *n* = 4; –endo, *n* = 4; PGE_2_, CTL, *n* = 10; AM237, *n* = 5; MAFP, *n* = 5; NS-398, *n* = 5; –endo, *n* = 7). (d) Schematic of the mechanism of TRPC5 regulation of endothelium-dependent contraction in the DIO mouse aorta. Mean ± SEM; a, *P < 0.05, NS, no significant difference *vs* NFD, Student's unpaired two-tailed *t* test; b, *P < 0.05 *vs* CTL, one-way ANOVA followed by Dunnett's multiple comparisons test; c, *P < 0.05 *vs* CTL, ^#^*P* < 0.05 *vs* AM237 group, one-way ANOVA followed by Turkey's multiple comparisons test.

As we had demonstrated the involvement of COX-2 in the TRPC5-regulated contractile response in DIO mice, we then investigated the involvement of EDCF candidates, the COX-2-derived prostanoid products. Five possible EDCF candidates, PGF_2α_, PGE_2_, PGD_2_, PGI_2_, and 8-isoprostanes were assayed using EIA kits. The ACh (10 μmol/L)-induced release of PGF_2α_ and PGE_2_, but not PGD_2_, PGI_2_, and 8-isoprostanes was higher in the DIO mouse aorta than in the NFD control (Fig. 7a). Importantly, the enhanced production of PGF_2α_ and PGE_2_ were inhibited by treatment with clemizole (20 μmol/L), knockout of TRPC5, treatment with MAFP, NS-398, and the removal of endothelium in the DIO mouse aorta (Fig. 7b). Beside, the AM237-elevated ACh-induced PGF_2α_ and PGE_2_ production was also markedly attenuated by treatment with MAFP, NS-398, and removal of endothelium in the NFD control mouse aorta (Fig. 7c). In sum, these data suggested that PGF_2α_ and PGE_2_ are involved in vascular contraction regulated by TRPC5-cPLA_2_ in DIO mice.

## 4 Discussion

The main findings of the present study are as follows: (1) TRPC5 plays a critical role in endothelium-dependent vascular contraction in obese mice. (2) Activated cPLA_2_ is involved in TRPC5-mediated contraction in the obese mouse aorta. (3) COX-2 contributes to the regulation of vascular contraction by TRPC5-cPLA_2_ under obese conditions. (4) PGF_2α_ and PGE_2_ are EDCFs released during vascular contraction regulated by TRPC5 under obesity. In sum, this study demonstrated a key role of the TRPC5-cPLA_2_-COX2-PGF_2α_/PGE_2_ pathway in vascular contraction under obese conditions (Fig. 7d).

Arterial endothelial cells are both the source and target of factors associated with cardiovascular disease, including obesity; they show an imbalance between beneficial EDRFs and harmful EDCFs [2,12,13]. Although endothelial TRPC5 has been shown to contribute to contraction in mouse carotid artery [9], its role in vascular tone control under pathophysiological conditions such as obesity remained elusive. Furthermore, the molecular mechanism of its contribution to obesity had not been determined. EDC plays a fundamental role in the maintenance and regulation of vascular tone and blood pressure [14]. Augmented EDC contributes to endothelial dysfunction and aggravates the progression of vascular diseases, including obesity [15]. An early signal in the EDC response is a cytosolic Ca^2+^ rise in endothelial cells, and this stimulates the production of contractile prostanoids, leading to vascular contraction [16]. A recent study showed that direct activation of the Ca^2+^-permeable ion channel TRPV4 induces endothelial-dependent contraction in the hypertensive mouse aorta [3]. However, very little was known about the molecular identity of endothelial Ca^2+^ entry channels that contribute to EDC in obesity.

The present study was designed to answer the following questions: (1) whether TRPC5 plays an essential role in the harmful effects of EDCFs to override its beneficial effects in obesity; and (2) whether the inhibition or knockout of TRPC5 ameliorates EDC in obesity. Our data revealed that TRPC5 expression is increased in DIO MAoECs. Activation of TRPC5 by the potent and selective pharmacological agonist AM237 markedly enhanced ACh-induced EDC in the NFD control mouse aorta, while pharmacological inhibition and genetic knockout of TRPC5 significantly ameliorated EDC in the DIO mouse aorta. We demonstrated that increased TRPC5 expression in obesity augmented the EDC response, which further promoted endothelial dysfunction. TRPC5 knockout and inhibition alleviated the endothelial dysfunction induced by obesity.

It has been reported that the phosphorylation of Ca^2+^-dependent cPLA_2_ is required for arachidonic acid release [17,18]. Arachidonic acid is metabolized by COX-1 and -2 to prostaglandins that are vasoconstrictor metabolites in endothelial and vascular dysfunction [16]. Our study suggested that the balance between vasodilator and vasoconstrictor metabolites of arachidonic acid is disturbed in obesity and is regulated by increased TRPC5 and activated cPLA_2_.

## 5 Conclusion

In sum, this study demonstrated that TRPC5 plays a role in vascular dysfunction in obesity by enhancing EDC in the DIO mouse aorta *via* activation of cytosolic cPLA_2_ in endothelial cells. We believe that this study establishes a generalized scheme for the role of different TRP channels in mediating EDC in different arteries in cardiovascular disease. Our study fills an important gap in the knowledge about EDC under disease conditions, and lays out a blueprint for the future development of TRPC5-based therapeutic options against obesity.

## Declaration of Competing Interest

The authors declare that they have no conflicts of interest in this work.

## Appendix Supplementary materials

Image, application 1
