# Supplementary material for: TRPC5 is essential in endothelium-dependent contraction of aorta from diet-induced obese mice
Source: Fundam Res. 2022 Jan 31;2(3):429–36. doi: 10.1016/j.fmre.2022.01.017 (PMC11197789; doi:10.1016/j.fmre.2022.01.017)
Supplement: Supplementary file 1 [file mmc1.docx]

**TRPC5 is essential in endothelium-dependent contraction of aorta in diet-induced obese mice**

Yifei Zhu^a,1^, Sheng Wang^a,1^, Yuan Chu^a^, Ka Zhang^a^, Xin Wen^a^, Lei Feng^a^, Fan Yu^a^, Xin Ma^a,^*

*^a^ Wuxi School of Medicine, Jiangnan University, Wuxi 214000, China.*

** Corresponding author: maxin@jiangnan.edu.cn (Xin Ma).*

^1^ *These authors contributed equally to this work*

***Keywords:*** transient receptor potential channel canonical family member 5 (TRPC5); endothelium-dependent contraction; vascular function; diet-induced obesity

### Supplementary figures

## *Supplementary figure 1*

**Fig. S1.** Genotyping of TRPC5 wild-type and TRPC5^-/-^ mice.

Representative PCR genotyping gel image of TRPC5^-/-^ and wild-type (WT) mice. Mouse tail genomic DNA was used. The sequences of the primers for TRPC5^-/-^ mouse genotyping were as follows: forward primer 1 (F1)- 5’GTAAGTGATACTAGGTATGGGGTATGGAGG, reverse primer 1 (R1)-5’GTCGACACACGTATAAGGCATACTCTTG 3’. The sequences of the primers for WT mouse genotyping were as follows: F1- 5’GTAAGTGATACTAGGTATGGGGTATGGAGG 3’, reverse primer 2 (R2)- 5’CTAACCATTCTTCTCACCTCTCTCTCCTC 3’. The F1/R1 primer combination generated an amplicon of 693 bp, and the F1/R2 primer combination generated one amplicon of 567 bp. The PCR conditions were as follows: 1) 95 °C for 2 min; 35 cycles of 2) 95 °C for 30 sec; 3) 58 °C for 45 sec; 4) 72 °C for 1 min; and a final step of, 5) 72 °C for 10 min.

## *Supplementary figure 2*

**Fig. S2.** Validation of AM237 compound efficiency in activating TRPC5 by patch clamp analysis. Representative current-voltage relationships (I-Vs) and data summary for basal and AM237-induced (100 nmol/L) currents in HEK and TRPC5-overexpressing HEK cells. Mean ± SEM of n = 3 independent experiments; **P* <0.05, NS, no significant difference *vs* baseline, Student’s unpaired two-tailed *t* test.

## *Supplementary figure 3*

**Fig. S3.** Validation of TRPC5 antibody specificity in WT and TRPC5^-/-^ mice. Representative western blot images (left) and data analysis (right) for anti-TRPC5 antibody in wild-type (WT) and TRPC5^-/-^ mouse aorta. n = 3. Mean ± SEM; **P* <0.05 *vs* WT, Student’s unpaired two-tailed *t* test.

### Supplementary table

## *Supplementary table 1*

**Table S1**

**Metabolic parameters of normal-fat diet (NFD) control and high-fat diet (HFD) induced obese mice**

|  | **NFD** | **HFD** |
| --- | --- | --- |
| Body weight (g) | 30.33 ± 0.33 | 42.01 ± 1.3 |
| Fat pad mass (% body weight) | 1.02 ± 0.23 | 4.71 ± 0.83 |
| Blood glucose (mmol/L) | 8.85 ± 0.61 | 13.94 ± 2.13 |
| Total cholesterol (mmol/L) | 3.98 ± 1 | 8.77 ± 0.96 |
| Triglyceride (mmol/L) | 1.08 ± 0.1 | 0.77 ± 0.28 |
| LDL cholesterol (mmol/L) | 0.55 ± 0.15 | 1.3 ± 0.16 |
| HDL cholesterol (mmol/L) | 1.3 ± 0.12 | 0.82 ± 0.13 |

NFD, n ≥ 6; HFD, n ≥ 6.

All statistical analyses were performed using the Student’s *t* test. The data are presented as mean ± SEM. A value of *P* < 0.05 was considered significant.

### Supplementary methods

#### Cell culture

Cells were cultured in Dulbecco’s modified Eagle’s medium supplemented with 10% FBS, 1% antibiotic-antimycotic and maintained at 37℃ with 5% CO_2_.

#### Whole-cell Patch clamp

Whole-cell patch clamping of MAoECs was conducted using an EPC10 patch clamp amplifier (HEKA, Holliston, MA, USA). Briefly, the pipette solution contained (in mmol/L): 130 Cs- aspartate, 2 MgCl_2_, 5 Na_2_ATP, 5.9 CaCl_2_, 10 EGTA, and 10 HEPES (pH 7.2) with CsOH. The bath solution contained (in mmol/L): 65 Na-aspartate, 5 KCl, 1 CaCl_2_, 1 MgCl_2_, 10 HEPES, 10 glucose (pH 7.4) with NaOH. The TRPC5 I-V relationship of primary aorta endothelial cells were obtained using a 500-ms ramp protocol from –80 mV to +80 mV from a holding potential of –60 mV. The cells were treated with AM237 (100 nmol/L) in the bath solution if necessary.

#### Western blot

Aorta tissue were lysed in lysis buffer (P0013C, Beyotime) on ice with protease and phosphatase inhibitors (Beyotime). The lysates were collected by centrifugation. The BCA assay was used for protein quantification. Proteins were electrophoresed on 10% SDS-polyacrylamide gels and then transferred to polyvinylidene fluoride membranes (Millipore Corp., Bedford, MA, USA). Skim milk powder (5%) was used for blocking. The membranes were incubated overnight at 4°C with the primary antibodies anti-TRPC5 (1:200, Proteintech) and anti-GAPDH (1:1000, Santa Cruz) followed by horseradish peroxidase-conjugated secondary antibodies (mouse, 1:10000; rabbit, 1:5000, Beyotime) at room temperature for 2 h. ImageJ was used for band intensity analysis.

#### Statistics

Data are represented as means ± standard error of the mean (SEM). Statistical analyses were performed by GraphPad Prism 6.0 software. Comparisons between two groups were analyzed by Student’s unpaired two-tailed *t*-test or the Mann-Wallis test. Differences among three or more groups were measured by one-way analysis of variance followed by Dunnett’s or Tukey’s multiple comparison test or Kruskal-Wallis and Dunn’s *post hoc* non-parametric test. *P*-values<0.05 were considered to be significantly different.
